# Histopathological study of cardiac lesions in methamphetamine poisoning-related deaths

**DOI:** 10.1186/s40199-017-0170-4

**Published:** 2017-02-17

**Authors:** Maryam Akhgari, Homeira Mobaraki, Afshar Etemadi-Aleagha

**Affiliations:** 1 Department of Forensic Toxicology, Legal Medicine Research Center, Legal Medicine Organization, Old Ghom Road, 500 meters after Beheste Zahra, 1816153141 Tehran, Iran; 2 Department of Forensic Pathology, Legal Medicine Research Center, Legal Medicine Organization, Tehran, Iran; 3 Tehran University of Medical Sciences (TUMS), Amir Alam Hospital, Tehran, Iran

**Keywords:** Methamphetamine poisoning-related death, Cardiac histopathology, Forensic toxicology, Forensic pathology, Forensic medicine

## Abstract

**Background:**

Methamphetamine abuse is a worldwide health concern. Methamphetamine causes health hazards in many vital organs. It can cause damage to cardiac tissue via catecholamines release. Methamphetamine related deaths are becoming one of the most important problems in Iran. The purpose of the present study was to determine cardiac pathology in methamphetamine poisoning-related deaths.

**Methods:**

The study included 100 cases of methamphetamine poisoning-related deaths and 100 cases as control group. Toxicology analysis of liver, gastric content, bile, urine, blood and vitreous humor were conducted to detect drugs, poisons and alcohols using thin layer chromatography, gas chromatography/mass spectrometry, and high performance liquid chromatography. Positive toxicology analysis results except for amphetamine and methamphetamine were excluded from the study in order to omit interfering factors. The most striking features of cardiac damage were observed by light microscopy.

**Results:**

Methamphetamine and amphetamine were detected in either urine or gastric content samples. In all of the cases methamphetamine toxicity was determined to be a direct cause of death by forensic medicine practitioner. Cardiovascular pathology was noted in 68% of studied cases. The most common histopathologic features were myocardial fiber hypertrophy, mild, moderate to severe atherosclerosis and focal degeneration/necrosis.

**Conclusion:**

The results of the present study indicate that cardiotoxicity is one of the major contributing factors in methamphetamine poisoning related deaths. Overall, the current study highlights the fact that cardiotoxic effects of methamphetamine can explain increasing reports of heart failure and consequently death in young abusers.

**Trial registration:**

Not applicable.

**Graphical Abstract:**

Histopathological study of cardiac lesions in methamphetamine poisoning-related deaths

## Background

Amphetamine type stimulants (ATS) are indirect sympathomimetic drugs that have been widely used to increase mood. Abuse of methamphetamine (MA) is a growing social problem all over the world. According to the World Drug Report, the global market for synthetic drugs continues to be dominated by MA. This report stated that Iran ranked fifth in MA seizure after Mexico, United States of America, China and Thailand [1]. This synthetic stimulant drug can be produced from inexpensive precursors. MA low cost production and long duration of action makes it an illegal substance with high abuse potential [2]. MA with the street name of “Shishe” has high prevalence of abuse in different population groups in Iran. MA high rate of domestic production and seizure confirms its widespread use. Although MA clandestine laboratories started illicit production since 1980s in Texas and some other countries [3], it was seized first in Iran since 2005. Till now it had been popular in young adults for different purposes. Polysubstance abusers, body builders and women use MA for increasing physical and social activities, enhancement of sexual performance and weight loss [4].

MA exerts its stimulant effect by induction of sympathetic nervous system activity and catecholamine overflow. It causes several central nervous and cardiovascular systems adverse effects through coronary vasoconstriction and the production of oxygen free radicals [5, 6]. MA causes a large number of problems in vital organs and no organ in the body remains unscathed by MA and its impurities [7]. Methamphetamine associated cardiomyopathy appears to be genetically dependent. Cytochrome p450 polymorphism is responsible for different grades of MA-induced cardiomyopathy. Individuals who are CYP2D6 extensive metabolizers are significantly vulnerable to MA-induced cardiomyopathy [8].

Alghamahdi et al. in a study conducted on patients admitted to a medical center in Saudi Arabia, found that the majority of cases with amphetamine abuse history showed cardiomyopathy, myocarditis and arrhythmia [6]. Kaye et al. showed coronary artery atherosclerosis, cardiomegaly and ventricular hypertrophy in MDMA (3,4- methylendioxymethamphetamine “ecstasy”) abusers in Australia [9]. Sadeghi et al. showed cardiomyopathy and congestive heart failure in three MA abuser cases admitted to Loghman Hakim Hospital, Tehran, Iran [10]. In a retrospective clinical study Wijetunga et al. reported that MA appears to produce cardiomyopathy in some users [11]. Previous case reports showed congestive cardiomyopathy, focal coagulation necrosis in myocardium, right ventricular rupture and arrhythmia in MA abusers who died of heart failure [5, 12, 13].

In spite of the introduction of novel psychoactive substances to drug black market all over the world, MA is the most abused substance in some African, American and Asian countries [2]. Therefore MA effect on vital organs has drawn a great deal of attention. MA abuse has appeared a new health problem in Iran [4]. Although there are many studies about psychiatric effects of ATS and their clinical complications in patients admitted to medical centers and also their adverse effects in experimental animal models, few reports exists on the histopathologic cardiac effects of MA abuse in forensic cases in Iranian population.

The aim of the present work was to study the toxicology and cardiac pathology of MA-poisoning related deaths in Iran.

## Methods

### Case selection

A total of 100 MA poisoning-related deaths occurring in Tehran, Iran with the age range of 21–35 years (young adult) were sequentially identified and selected over one year study period (January 1^st^, 2015 till December, 30^th^ 2015). In order to reduce bias, all cases with a history of previous cardiovascular complications were not included in the present study. As MA abuse is prevalent in young age ranges, study cases were selected from this range of ages. Also 100 age and sex-matched MA negative control group were selected from cases with no history of substance abuse. The manner of death of control cases was car accident, falling down or stab wound. All cases with positive toxicology results for drugs, alcohols and poisons other than MA were excluded from the study (56 cases) due to the probable toxicity induced histopathological changes in selected organs such as heart.

### Chemicals and reagents

Methamphetamine hydrochloride (HCl) was purchased from Lipomed Pharmaceutical (Arlesheim, Switzerland). Water, n- hexane, n-heptane (high-performance liquid chromatography (HPLC) grade), formalin, xylol, hematoxylin and eosin were purchased from Merck Company (Germany). Potassium hydroxide (KOH) and sodium bicarbonate (NaHCO_3_) were Reagent Plus®, 99.9% and were supplied by Sigma Aldrich (Germany). Heptafluorobutyric acid (HFBA) in derivatization grade was obtained from Sigma Aldrich (Germany). Paraffin was purchased from Sakura Company, Japan.

### Toxicology analysis

In situations of unnatural deaths especially in young cases, qualitative and quantitative toxicological analysis is necessarily and routinely performed to detect the cause of death. Samples collected at autopsy examination are looked for drugs according to the toxicology laboratory protocols. Thin layer chromatography (TLC), headspace gas chromatography (HSGC), Agilent 6890 N (USA) model equipped with flame ionization detector, with chromatographic column DB-ALC1, capillary column (30 m × 0.320 mm × 1.8 mm), high performance liquid chromatography (HPLC) (Knauer, Germany) with a diode array detector (DAD) (Knauer DAD 2700, Germany) equipped with a quaternary pump (Knauer pump 1000, Germany) and gas chromatography (GC) (Agilent Technologies, sdn Bhd, Selangor) and mass spectrometry (MS) 5975C model (Agilent Technologies) were used to perform toxicology tests on liver, urine, blood, vitreous humor, gastric content and bile. Predeveloped method was used to extract MA and amphetamine (AM) using liquid liquid extraction (LLE) technique. Urine or stomach content samples were vortexed and centrifuged for 5 min at 9000 r/min. Separated supernatants were transferred into a glass tube. After the addition of KOH (0.5 M), pH was adjusted to 11–12. Desired analyte (methamphetamine) was extracted by adding n-hexane and n-heptane. HFBA was used as derivatization reagent. Excess amount of HFBA was deactivated by adding NaHCO_3_. Organic layer was separated and analysed. GC/MS instrumentation was used to detect MA and AM. The method was fully validated and showed acceptable intra and inter assay precision. Limit of detection (LOD) and limit of quantitation (LOQ) for MA were 5 and 15 ng/mL respectively.

GC method chromatographic conditions were as following:

Helium (99.999%) with 1.5 mL/min constant flow rate, inlet temperature 250 °C and injection volume of 1 μL (splitless) were used. The oven temperature was set at 90 °C (held for 1 min) followed by 20 °C/min ramp to 280 °C and held for 5 min. Mass source and quadrupole temperatures were set at 230 °C and 150 °C, respectively. The ion source was operated in full scan and selected ion monitoring (SIM) mode both together. In full scan mode, scan range was 40–500 m/z, ions selected for quantitative analysis were 118 and 254 for MA.

### Histologic examination

Histologic examination was performed on cardiac tissue of MA poisoning-related death cases that had already been confirmed by analytical toxicology results. The cause of death of all cases was determined as MA poisoning by forensic practitioners.

Pre-established macroscopic and histological criteria were used for the diagnosis of cardiac pathology. The histopathological assessment included the inspection of ventricles, pericardium, endocardium, and coronary arteries. Each sample was scored histopathologically with a grade of 0 = negative, 1 = mild (occasional scars), 2 = moderate (multiple scars), or 3 = severe (extensive) to evaluate the extent of heart histopathological changes. Cardiac tissues of 100 control and cases were formalin-fixed. After fixation, samples were dehydrated in alcohol series and embedded in paraffin wax in order to be stained by hematoxylin and eosin and examined under light microscopy for histopathological study based on Kiernan protocol [14]. Two pathologists reviewed slides blindly. Any gross and microscopic features of cardiac tissue were considered and histologic changes for each case were recorded.

### Statistical analysis

Quantitative variables were expressed as mean ± SD, and qualitative variables were expressed as percentages. Descriptive statistics were used to describe the basic features of the data in the study. Also logistic regression analysis was used to determine the association between MA toxicity and cardiac histology. In order to determine the effect of some risk factors in general population and the presence of cardiovascular pathology, multivariate logistic regression analyses were conducted with independent variables such as gender, age and body mass index (BMI). Results presented as 95% confidence intervals (CI) and odds ratio (OR). *P* values < 0.05 and 95% CI which do not include OR = 1 are considered significant.

### Ethics approval and consent to participate

According to Legal Medicine Research Center, Iran’s ethics Committee, the information about human cadavers was fully confidential. Data were anonymously treated. The project was approved with the registration number: 1588. The study protocol conformed to the ethical guidelines of 1975 Declaration of Helsinki, as revised in 1983.

## Results

To detect the cardiac histology changes of MA poisoning-related deaths, we examined biological samples obtained at autopsy examination from toxicological and pathologic point of view.

### Demographic characteristics

In the present study methamphetamine was noted as a direct cause of death in all 100 cases. The mean age (standard deviation) of cases was 28.2 (2.80) years with the age range of 21–35 years. The majority of cases were men (95%). Less than 30% of cases were in the treatment program for drug dependence at the time of death. The mean BMI (standard deviation) of cases was 26.7 (4.32). Males showed significantly higher BMI than females (*p* < 0.05).

### Toxicological analysis results

**Fig. 1:**
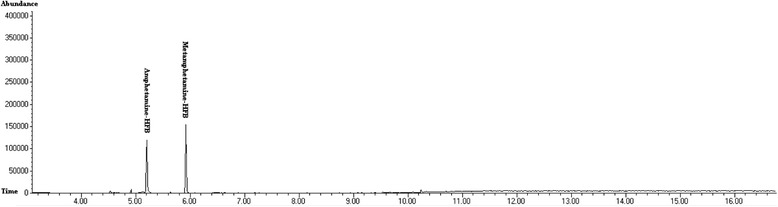
Chromatogram of derivatized amphetamine and methamphetamine with heptafluorobutyric acid detected in the urine sample of a fatal methamphetamine-poisoning related death case

**Fig. 2:**
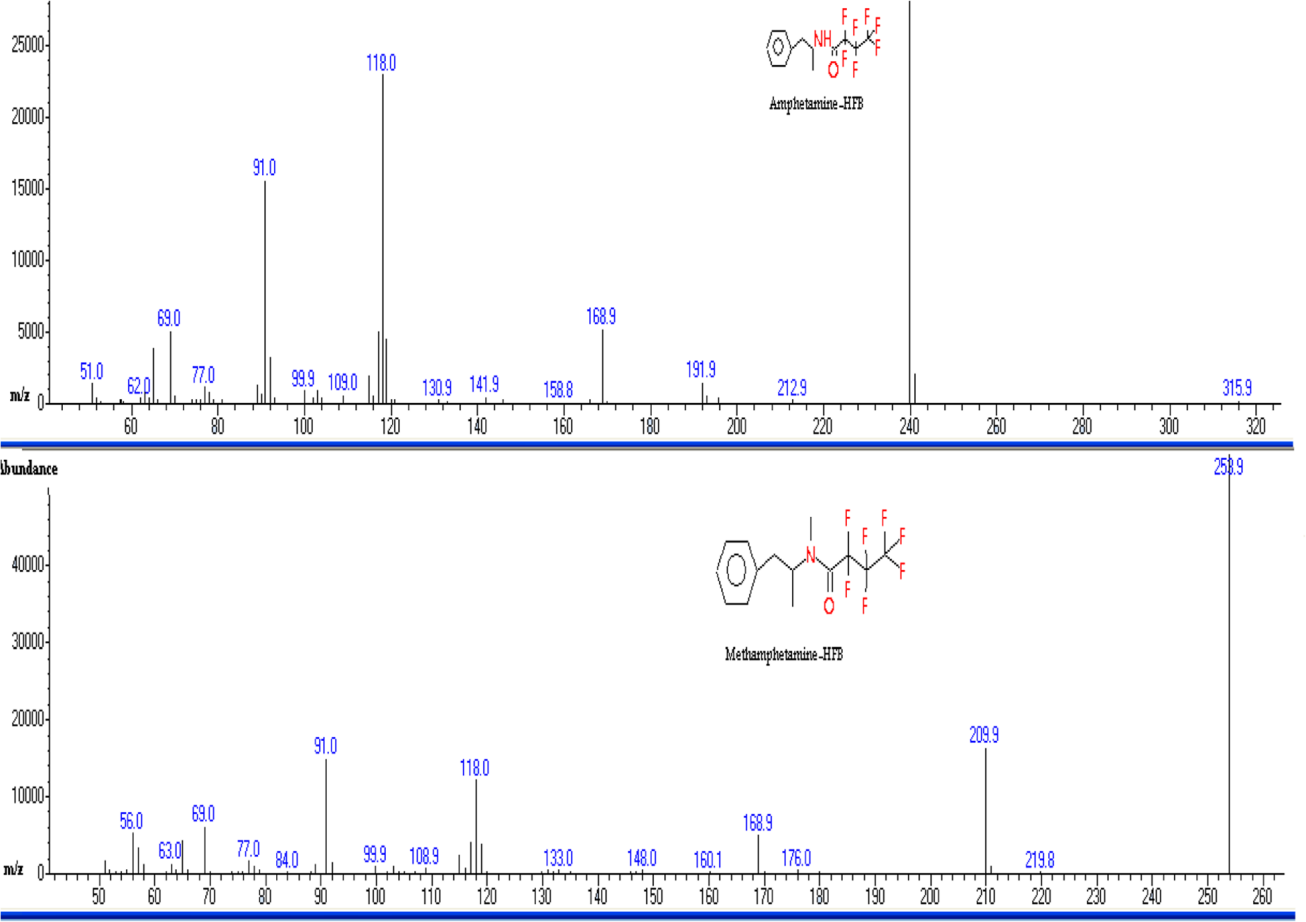
Mass spectra of derivatized amphetamine and methamphetamine with heptafluorobutyric acid detected in the urine sample of a fatal methamphetamine-poisoning related death case

Vitreous humor and blood samples were negative for alcohols (methanol, ethanol and isopropanol). Urine, liver, gastric content and bile samples were negative for drugs and poisons other than methamphetamine except for excluded cases. Methamphetamine and its metabolite, amphetamine were detected in urine or gastric content samples. Figures 1 and 2 show chromatogram and mass spectra of derivatized amphetamine and methamphetamine with heptafluorobutyric acid in the urine sample of a fatal methamphetamine-poisoning related death case.

All of the samples were negative for other conventional, illegal drugs and also poisons.

Noticeable in our study was the high percentage of cases (56 cases) with multiple drug intoxication (especially opium alkaloids, methadone, tramadol and ethanol). These cases were excluded from the study.

### Histologic examination results

The gross examination of the heart showed varied from no structural changes to pattern of dilated cardiomyopathy.

**Fig. 3:**
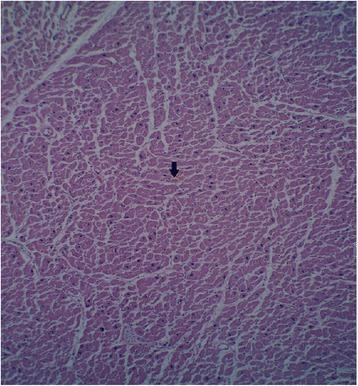
Histopathological section of myocardial fiber hypertrophy in a methamphetamine poisoning-related death case. *Arrow* shows scattered enlarged dark nuclei in hypertrophic myocardial fiber. Hematoxylin and eosin stain, 40× original magnification

**Fig. 4:**
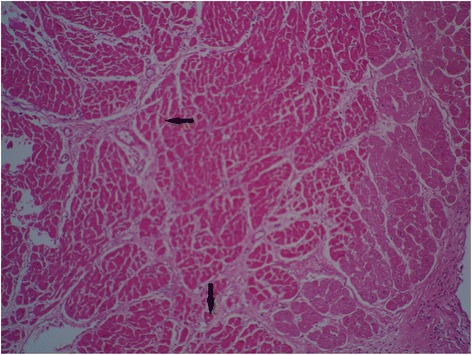
Interstitial and perivascular fibrosis in myocardium of a methamphetamine poisoning-related death case showed by *arrow*. Hematoxylin and eosin stain, 40× original magnification

**Fig. 5:**
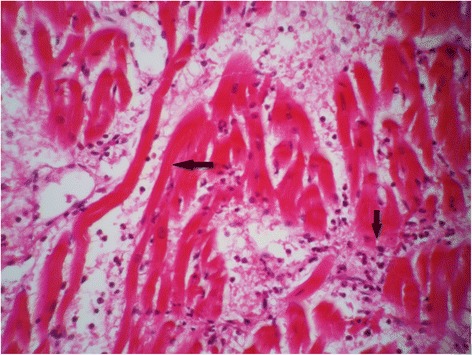
Acute myocardial infarction in a methamphetamine poisoning-related death case. *Arrows* show edema, fiber necrosis and polymorphonuclear (PMN) infiltration. Hematoxylin and eosin stain, 100× original magnification

**Fig. 6:**
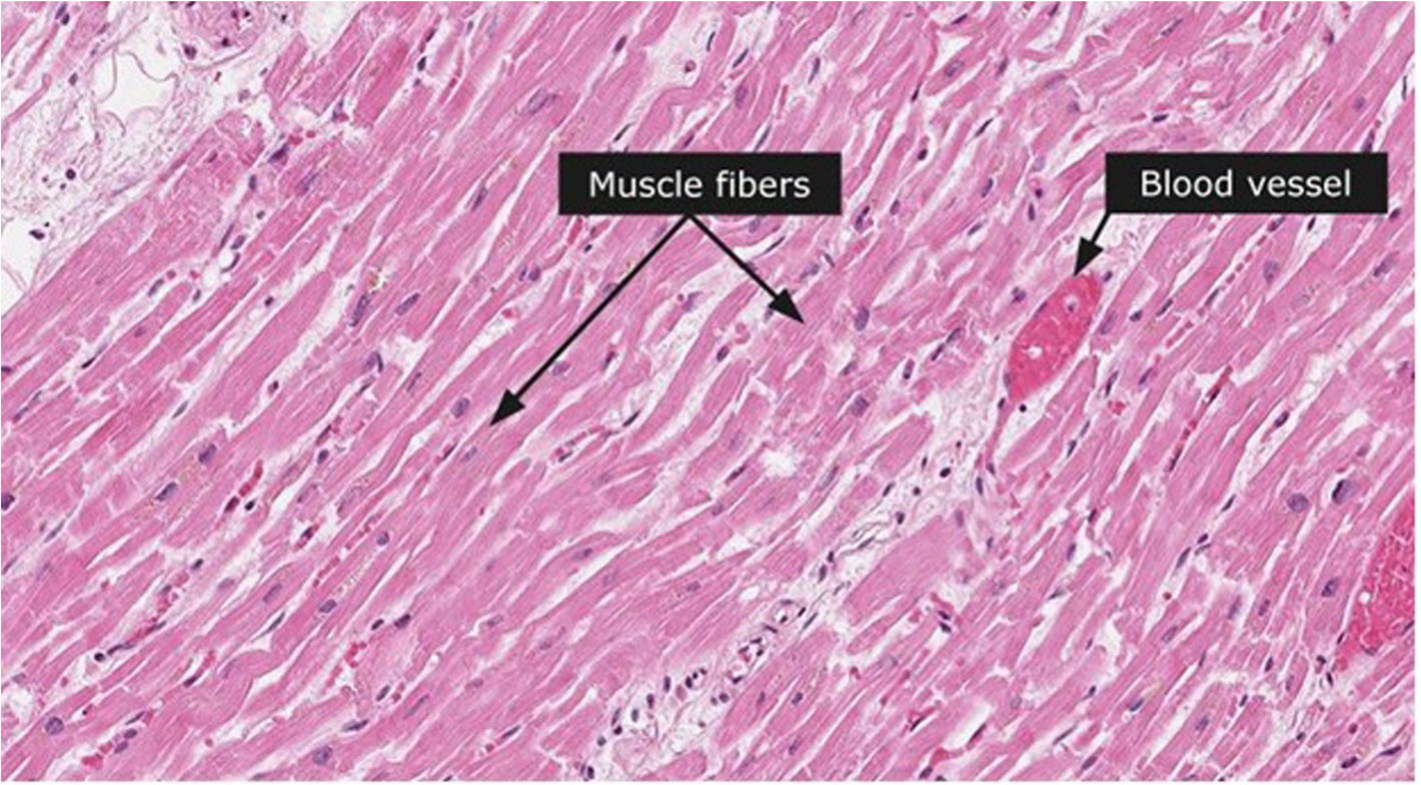
Histologic section of a normal myocardium of a control case. *Arrows* shows normal muscle fiber and blood vessel. Hematoxylin and eosin stain, 40× original magnification

Full autopsy reports of all 100 control and 100 case subjects were available to the forensic pathologists. Cardiovascular pathology was noted in 68% of cases. Some commonly histopathologic feature was myocardial fiber hypertrophy (Fig. 3), perivascular fibrosis (Fig. 4) and acute myocardial infarction (Fig. 5). Figure 6 shows the normal cardiac tissue of a control subject.

Table 1 shows a summary of the main patterns of morphologic cardiac injury in 100 cases of MA poisoning-related deaths. It should be taken into account that coexistence of two or three cardiac histology changes in one case is common.

**Table 1:** Major cardiac histopathology findings in methamphetamine poisoning-related death cases (*n* = 100) by light microscopy

Cardiac pathology feature	Frequency (%)
Mild atherosclerosis	22
Moderate to severe atherosclerosis	17
Congestion	22
Microscopic hemorrhage	7
Myocardial fiber hypertrophy	24
Perivascular fibrosis	9
Focal degeneration/necrosis	13
Myocardial ischemia	8
Acute myocardial infarction	3
Old subendocardial myocardial infarction	2
Mural thrombosis	1
Endocarditis	1
Pericarditis	4
None	32

The presence of cardiac tissue damage however was not significantly predicted by any of the aftermentioned variables (age, sex and BMI). Methamphetamine abuse was significantly associated with cardiovascular pathology (95% CI, OR = 1.7, *p* < 0.05).

## Discussion

The purpose of the present study was to investigate the effect of MA on cardiac tissue in MA poisoning-related death cases. According to the results of the study MA has contributed to clinically significant histologic changes in cardiac tissue. MA was a direct cause of death in all studied cases. These results were consistent with previous studies in cases of ATS related deaths [9, 15, 16]. Decedents were in the young age range, a demographic profile similar to other studies [9]. In consistent with the results of the present study, several epidemiological studies in Iran demonstrated that more than 90% of illicit drug users were men [4, 17, 18]. Although MA use treatment programs have been provided by public and private centers under the authority of the Ministry of Health and Medical Education, Iran, less than 30% of our study cases were referred from these centers. This indicates that in spite of severe control, abusers have free access to illegal drugs in treatment centers.

MA poisoning was noted as a direct or antecedent cause of death. Cardiac histology changes were correlated with MA abuse independent to variables such as gender, age and BMI. These results are in agreement with those of Kaye et al. who studied on methylendioxymethamphetamine related fatalities in Australia [9].

Methamphetamine can be detected in various biological matrices in forensic and clinical laboratories [19]. Comprehensive toxicology testing revealed the presence of no drugs with the exception of MA and AM in all cases in the present study.

To our knowledge this study is the first to report cardiac histology changes in MA poisoning-related deaths in Iranian population except for a few case reports that had been published in regard to MA induced cardiomyopathy in clinical cases not in forensic ones [10, 20, 21]. The present study aimed to address this gap in Iran.

There are genetic variations in metabolism and pharmacologic response to drugs. In this regard drug related deaths could be interpreted with difficulty in forensic cases [22].

Methamphetamine undergoes three metabolic pathways in the body: 1) n-demethylation reaction to produce amphetamine, 2) aromatic hydroxylation making 4- hydroxyamphetamine (pholedrine), 3) beta- hydroxylation producing norephedrine [23–25]. The polymorphic cytochrome P450 isozyme, CYP2D6 is responsible for the metabolism of most ATS. Acute toxicity, dependence and long-term neurotoxicity could be influenced by CYP2D6 genetics [23]. Catalytic activity of CYP2D6 varies considerably in different populations as a result of genetic variation. It is assumed that Asian population are slower in the rate of metabolic disruption of CYP2D6 substrates, leading to a higher tendency to accumulate some drugs such as MA in the body [23]. It is supposed that genetic variation in Iranian population can cause different responses to MA. Sutter et al. in their study concluded that the polymorphism in CYP2D6 enzyme may predict MA-related heart complications [26].

There are some reports that ATS can cause tissue damage in vital organs through different mechanisms [12, 27, 28]. The mechanisms of producing cardiomyopathy in MA animal and human users are multifactorial. Suggested mechanisms for MA-induced cardiac injuries are: excess of catecholamines, coronary vasospasm and ischemia, reactive oxygen species production, mitochondrial dysfunction, accelerated apoptosis, cardiomyocytes necrosis, defects in intracellular calcium hemostasis and abnormality in cardiac protein production [29, 30]. The pattern described in the present study overlaps with the histological picture seen in previous studies. Myocardial fiber hypertrophy and myocardial ischemia were seen in 24 and 8% of our cases respectively. As it is shown in Fig. 3 in hypertrophic cardiomyocytes nuclei are enlarged. As a consequence of increased amount of their constituent DNA, appear hyperchromatic [31]. Ischemia can be seen in hypertrophic hearts. In spite of the promotion of vascularization with expanding muscle mass, oxygen and nutrients cannot be carried completely through the microvascular system to reach myocytes. Therefore in conditions such as exercise, increased heart rate and arrhythmia, myocardial ischemia may be triggered [12, 27]. It seems likely that hypertrophy is one of the diagnostic factors for MA-related deaths from heart failure [5]. The results mirror exactly those of the Rosenblum et al. survey, which reported that drugs that can release heart catecholamines produce cardiac lesions in experimental animal models. Chronic elevation of catecholamines causes cardiac remodeling and also hypertrophy and fibrosis [32]. Positive inotropic (amphetamines) and chronotropic substances increase oxygen consumption, local hypoxia and subsequently cell necrosis [28]. Perivascular fibrosis was noted in 9% of cases (Fig. 4). This result is in agreement with those of Karch who reported perivascular fibrosis in a case of MA-related death. Perivascular fibrosis reduces myocardial flow reserve and possibly generates myocardial infarction [12].

In accordance with previous research [2, 9], mild and moderate to severe atherosclerosis was typically seen in the coronary arteries. MA induces serotonin release from nerve terminals and MA cardiotoxicity is held by oxidative damage with underlying oxidative stress, consequently it may have a key role in the pathophysiology of atherosclerosis [33, 34]. In the present study three cases showed acute myocardial infarction. Accelerated atherosclerosis and rupture of preexisting atherosclerotic plaques are some features inducing myocardial infarction in MA abusers [35]. Also Gao et al. reported that chronic MA administration to animal models is accompanied by inflammation and atherosclerotic plaque formation [36].

Moreover Hosseini-Sharifabad et al. in a study conducted to evaluate the effect of ecstasy on mouse cardiac histopathology found that increased levels of some neurotransmitters (serotonin, dopamine and norepinephrine) can cause adverse cardiovascular effects [37].

Some of our findings do not support those of Islam et al. in that; they found no histologic evidence of edema and fibrosis in the cardiac tissue of rats treated with MA [38]. However in the present study in pathologic examination enlarged, dilated ventricles and atria and congestion were observed in 22% of cases. Several aspects may help explain the discrepancy between results. Their research was undertaken on experimental rat models and our results obtained from human MA poisoning-related death cases. They had used pure methamphetamine with known doses to induce cardiac lesions, yet our study cases had used different types of impure MA with undefined doses. The required and enough dose of MA for producing cardiovascular complications or even death are not clear. Not all people respond to a specific dose of MA in the same manner due to interindividual variations in degree of tolerance and response to MA. Therefore it is not reasonable to estimate MA toxicity based on its dose [39]. Besides impurities in street drugs can cause health hazards [7]. In Iran, street MA (Shishe) is an impure cocktail of different chemicals originated from manufacturing processes and other active pharmaceutical ingredients with unknown doses [7, 40, 41]. In previous studies conducted on street MA samples in Iran many impurities of origin such as N-acetylmethamphetamine, N,N-dimethylamphetamine, phenmethrazine and benzaldehyde were detected [7, 42, 43]. Cardiotoxic effects of phenmetrazine were discussed in previous studies. In severe poisoning phenmetrazine causes cardiovascular collapse, myocardial ischemia, ventricular dysfunction and infarction [7]. It should be noted that none of the active pharmaceutical ingredients other than methamphetamine and amphetamine were detected in biological samples in the present study.

Intravenous drug abusers suffer from cardiac infections, endocarditis and pericarditis [42–44]. Although it was reported that bacterial endocarditis and pericarditis are rare [45], this study showed that one and four percent of study cases had infective endocarditis and pericarditis respectively. Besides mural endocardium thrombosis was observed in combination with endocarditis. Illicit drugs are produced in substandard conditions and diluted several times along distribution lines by dealers. Bacterial spores can be introduced into the drug during these processes and cause infectious diseases such as endocarditis, osteoarthritis and hepatitis [46, 47]. Tamizifar et al. reported acute purulent pericarditis caused by Klebsiella Pneumoniae in a 30-year old opioid abuser [48]. Pericarditis due to Bacillus Cereus was reported in an IV drug user [49]. MA injection is a new emerging health problem in Iran. In a survey of 209 MA injection abusers, 47.4% reported that they use sharing syringes when injecting MA [4]. Therefore it is logical to see different results from other studies due to higher prevalence of using common syringes for injecting drugs.

The present study is limited in several ways. Dose and type of abused MA was not known and unequal in all of the cases. Duration and history of substance abuse habit were not defined. However as cardiovascular complications in MA abusers can be produced in low doses, this limitation did not overshadow our main purpose. A large number of samples were excluded from the study as a result of the positive outcomes obtained at toxicologic examination for other drugs and abused substances (opium alkaloids, methadone, tramadol and ethanol). The other limitation of course is the lack of knowledge of previous drug use.

## Conclusion

There is growing body of evidence that MA abuse can cause major health problems in vital organs and consequently death. Mild, moderate-severe hypertrophy, myocardial fiber hypertrophy and other cardiac pathology features were observed in MA-related deaths irrespective of age, sex and BMI. This recognition may prove to be of some forensic value to clarify the cause of death in MA abusers.
